# Bridging the cervicothoracic junction during posterior cervical laminectomy and fusion for the treatment of multilevel cervical ossification of the posterior longitudinal ligament: a retrospective case series

**DOI:** 10.1186/s12891-022-05417-3

**Published:** 2022-05-12

**Authors:** Dong-Zhao Wu, Zhen-Fang Gu, De-Jing Meng, Shu-Bing Hou, Liang Ren, Xian-Ze Sun

**Affiliations:** 1 Department of Spine Surgery, The Third Hospital of Shijiazhuang, No. 15 Tiyu Street, Shijiazhuang, 050000 China; 2 Emergency Follow-up Department, Shijiazhuang Emergency Center, Shijiazhuang, No. 188 Jianshe Street, 050000 China

**Keywords:** Ossification of the posterior longitudinal ligament, Laminectomy, Cervicothoracic, Fusion

## Abstract

**Background:**

The purpose of this study was to investigate the surgical efficacy of crossing the cervicothoracic junction during posterior cervical laminectomy and fusion for the treatment of multilevel cervical ossification of the posterior longitudinal ligament (OPLL).

**Methods:**

From October 2009 to October 2017, 46 consecutive patients with multilevel cervical OPLL underwent posterior cervical laminectomy and crossing the cervicothoracic junction fusion were obtained in the study. Their medical records were retrospectively collected. Cervical lordosis and cervical sagittal balance were used to assess radiographic outcomes. Japanese Orthopedic Association (JOA), axial symptom, C5 root palsy, blood loss, and operation time were used to assess clinical outcomes. The mean follow-up period was 20.7 ± 8.3 months.

**Results:**

The operation time was 205.2 ± 39.8 min and the intraoperative blood loss was 352.2 ± 143.7 ml. Analysis of the final follow-up data showed significant differences in JOA score (*P* < 0.01), C2-C7 lordosis angle (*P* < 0.01), and C2-C7 SVA (*P* < 0.01). CT confirmed that grafted bone was completely fused in all patients and progression of OPLL was observed in two patients (4.3%) at final follow-up. No adjacent segment disease (ASD) or instrument failure occurred in any patients.

**Conclusions:**

Cervical laminectomy and crossing the cervicothoracic junction fusion are effective and safe methods to treat multilevel cervical OPLL. Randomized controlled studies compared constructs ending at cervical vertebrae or thoracic vertebrae are needed to confirm these results.

## Background

Ossification of the posterior longitudinal ligament (OPLL) is attributed to heterotopic ossification of the cervical or thoracic PLL, potentially leading to spinal cord compression and neurologic deterioration [1, 2]. At a mean follow-up of 17.6 years, only 17% of 323 patients without myelopathy evident at the first examination developed myelopathy [3]. So it’s neither necessary nor recommended to perform prophylactic surgery [1]. However, in patients with myelopathy evident at the first examination who underwent conservative therapy, 64% had deteriorated and 89% of patients with Nurick grade 3 or 4 myelopathy managed conservatively became completely disabled [3]. In addition, Cervical spinal cord injury and related disability are more likely to occur in OPLL patients [4]. For these reasons, surgical decompression is considered in patients with progressive myelopathy.

Surgical options include anterior corpectomy and fusion, laminoplasty, and laminectomy and fusion [5]. All surgical methods have advantages and disadvantages. Currently the optimal surgical treatment of cervical myelopathy caused by OPLL remains controversial [1]. The purpose of this retrospective study was to evaluate the surgical efficacy of crossing the cervicothoracic junction during posterior cervical laminectomy and fusion for the treatment of multilevel cervical OPLL.

## Methods

### Patients

From October 2009 to October 2017, 46 consecutive patients (38 males, 8 females; mean age 55.4 ± 7.4 years) with multilevel cervical OPLL underwent posterior cervical laminectomy and crossing the cervicothoracic junction fusion were obtained in the study. Their medical records were retrospectively collected. Patient inclusion criteria were (1) radiographic confirmation of cervical OPLL, (2) compressive lesion more than 3 cervical levels, and (3) clearly documented progressive cervical myelopathy. Exclusion criteria were (1) fractures, tumors, and metabolic disorders, (2) only axial neck pain without myelopathy, (3) concurrent anterior cervical spine procedures, and (4) prior surgery of the cervical spine.

### Operative technique

In our center, all spinal surgeries were performed under monitoring of transcranial motor-evoked potentials, somatosensory-evoked potentials, and free-running electromyography. The patients were placed in the prone position after general anesthesia. A standard posterior midline exposure was performed for all procedures. The paravertebral muscles were retracted laterally and muscle insertion of C2 spinous process was retained. Then, C2 was implanted pars screws. Lateral mass screws were placed from C3 to C5. Pedicle screws were placed in C7 and T1. We did not implant screws in C6 to facilitate installation of connecting rods (Fig. 2 c and d). Subsequently, A dome-shaped sublaminar decompression was performed at C2 with a high-speed burr. From C3 to C7, a full-thickness trough was drilled at the junction of the lateral mass and the lamina with a high-speed burr. The laminae were elevated from the one side toward the other side and were removed completely. Then, enlarged laminectomy was performed to ensure adequate decompression which including adequate decompression of neural foramina and the removal of the inside edges of facet joints. Finally, posterolateral bone grafting at the fixation region was performed. Postoperatively, patients were required to stay in bed for 3–5 days and thereafter walking was allowed with a cervical collar for one month.

### Radiologic and clinical evaluation

**Fig. 1:**
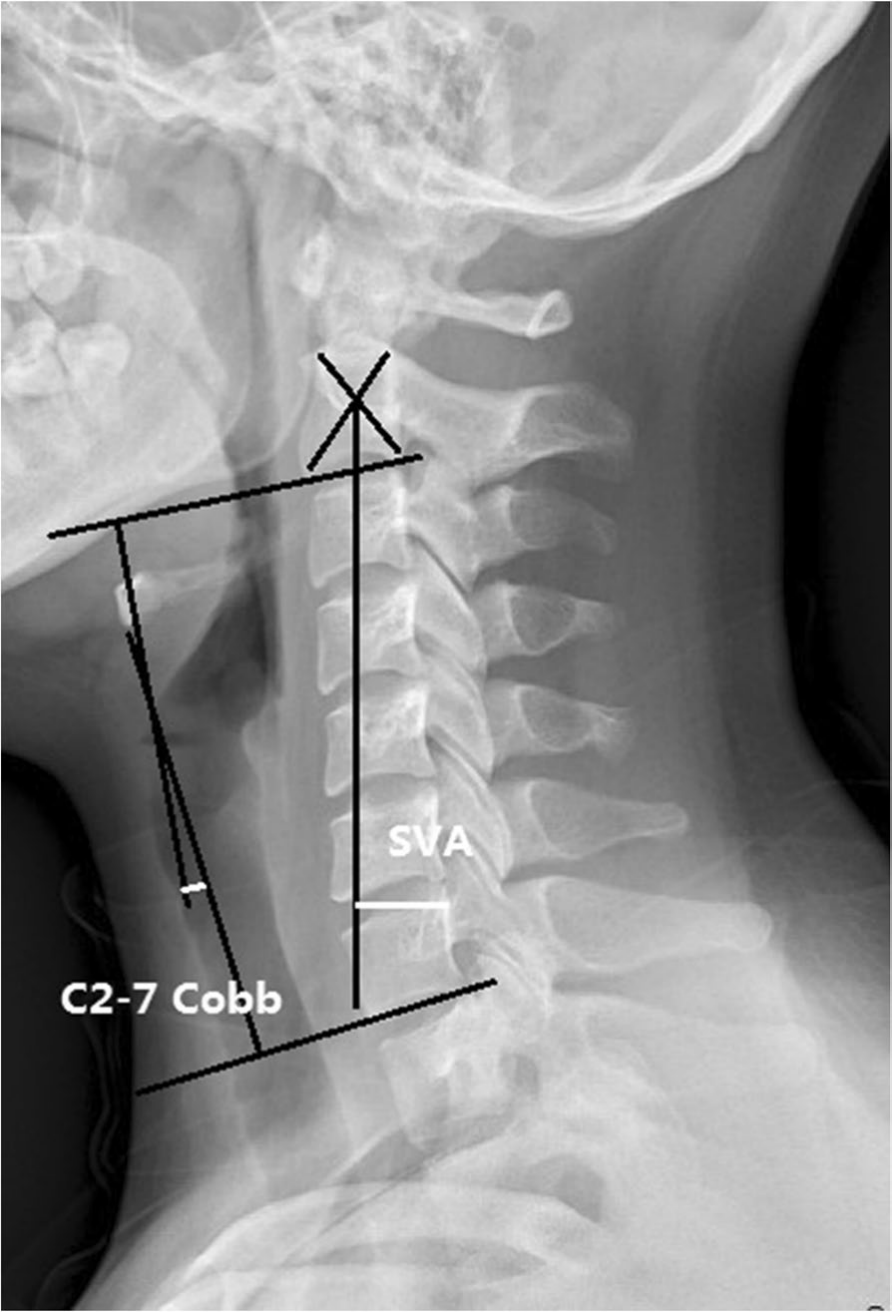
The evaluation of the C2–C7 Cobb angle and the SVA

**Fig. 2:**
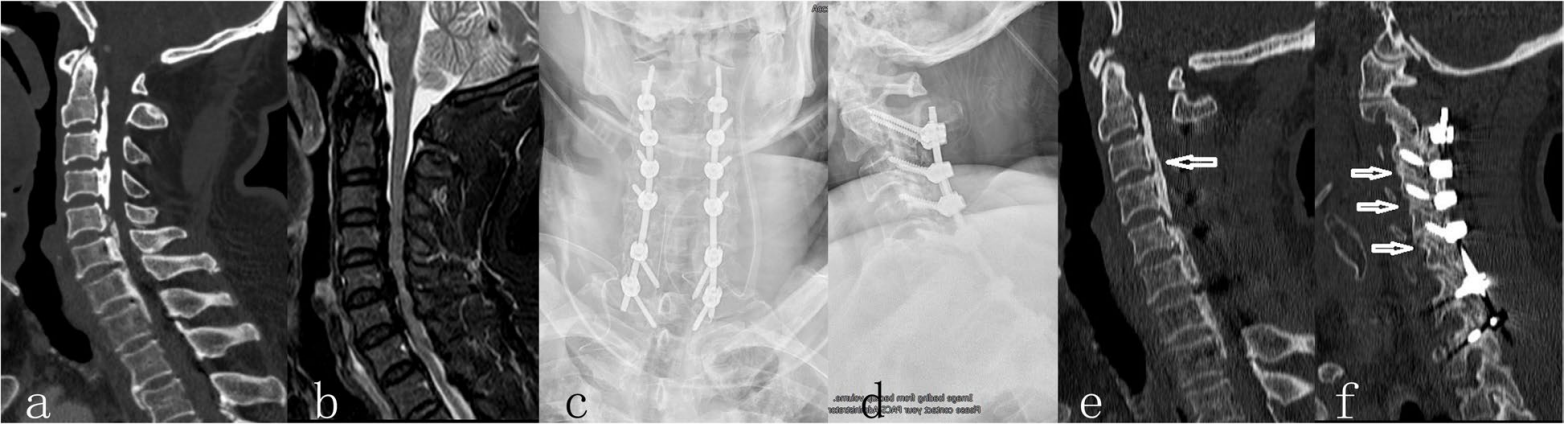
A 63-year-old male patient with multilevel, mixed-type ossification of the posterior longitudinal ligament. Preoperative computed tomography scan showed ossification of the posterior longitudinal ligament involving C2-T1 (**a**). Preoperative magnetic resonance image showed the pathological extent involving 6 intervertebral levels (**b**). He accepted crossing the cervicothoracic junction fusion from C2 to T1 and acquired sufficient decompression from C2 to C7 (**c**, **d**). Computed tomography scan of 12 month after surgery showed progression of ossification of the posterior longitudinal ligament (**e**). Computed tomography scan of 12 month after surgery showed fusion in the facet joints (**f**)

Radiographs, computerized tomography (CT), and clinical evaluation was performed preoperatively and at the final follow-up. Type of the OPLL was classified as localized, segmental, continuous or mixed morphology basing on the sagittal CT images (Fig. 2 a). The cervical sagittal balance was measured by C2-C7 lordosis and C2-C7 sagittal vertical axis (SVA) as shown in Fig. 1. C2-C7 lordosis was defined as the sagittal Cobb angle between C2 and C7 vertebral bodies. C2-C7 SVA was defined as the distance between C2 plumbline and C7. OPLL progression was measured by length and depth growth [6]. A three-dimensional CT scan was performed to confirm fusion and OPLL progression. Progression of OPLL was defined as an increased ossification of ≥ 2 mm in length or thickness [7]. Kyphosis line (K-line) was defined as a line connecting the center of the canal at C2 to the center at C7 on neutral radiographs. The K-line (-) was defined when the OPLL exceeded the K-line and K-line ( +) was defined when the OPLL did not exceed the K-line (Fig. 2 b). The OPLL occupying ratio (OR) was defined as the biggest ratio of OPLL thickness to antero-posterior diameter of the bony spinal canal on the axial CT image. All radiological evaluations were performed by an independent surgeon who was not involved in patient treatment.

Clinical outcome was measured by Japanese Orthopedic Association (JOA) scoring system. The neurological recovery rate was calculated as (postoperative JOA score-preoperative score)/(17-preoperative score)*100%. Recovery rates were graded as follows: 75% and greater, excellent; 50 to 74%, good; 25 to 49%, fair; and less than 25%, poor [8]. Blood loss, operation time, and complications were reviewed for each case.

### Statistical analysis

We used the SPSS statistical package (version 21.0; SPSS Inc, Chicago, IL, USA) for data analysis. *P* < 0.05 was considered significant. A paired t test was used to assess statistical significance of changes between postoperative and preoperative parameters in each group.

## Results

### Patient characteristics

Posterior cervical laminectomy and crossing the cervicothoracic junction fusion were performed in all patients. The patient characteristics were summarized in Table 1. The mean symptomatic duration was 34.9 ± 12.3 months. In total, 5 patients (10.9%) had segmental OPLL, 27 patients (58.7%) had continuous OPLL and 14 patients (30.4%) had mixed OPLL. Surgical extent was from C2 to T1 in all patients. The average follow-up period was 20.7 ± 8.3 months (range 12–48 months). The average operation time was 205.2 ± 39.8 min (range 120–300 min) with a mean intraoperative blood loss of 352.2 ± 143.7 ml (range 150–800 ml).

**Table 1:** Patient characteristics

Characteristics	Value
Number of patients	46
Age (years)	55.4 ± 7.4
Gender	
Male	38
Female	8
Symptomatic duration (months)	34.9 ± 12.3
High-intensity signal on T2WI-MRI	12
Type of OPLL	
Local	0
Segmental	5
Continuous	27
Mixed	14
Occupying ratio of OPLL	
< 60%	36
≥ 60%	10
K-line	
( +)	31
(-)	15
Follow-up (month)	20.7 ± 8.3
Operation time (minute)	205.2 ± 39.8
Blood loss (ml)	352.2 ± 143.7

*OPLL* Ossification of the posterior longitudinal ligament

### Neurological results

The JOA scores had significantly improved from preoperative 8.0 ± 2.0 to 13.8 ± 1.8 at final follow-up (*p* < 0.01) (Table 2). The average neurological recovery rate was 65.6 ± 14.2%. Neurological recovery rates were excellent in 16 (34.8%) patients, good in 25 (54.3%) patients, fair in 5 (10.9%) patients, and poor in 0 (0%) patients.

**Table 2:** Summary of clinical and radiologic outcomes

*n* = 46	Preoperative	Final	P
JOA score	8.0 ± 2.0	13.8 ± 1.8	< 0.01
C2-C7 lordosis angle (°)	6.0 ± 3.1	9.5 ± 3.0	< 0.01
C2-C7 SVA(mm)	26.7 ± 4.9	25.4 ± 4.3	< 0.01

### Radiological results

X-ray radiographs showed that C2-C7 lordosis angle had significantly increased from 6.0 ± 3.1° preoperatively to 9.5 ± 3.0 at final follow-up (*p* < 0.01) and C2-C7 SVA had significantly decreased from 26.7 ± 4.9 preoperatively to 9.5 ± 3.0 at final follow-up (*p* < 0.01). CT confirmed that grafted bone was completely fused in all patients and progression of OPLL (Fig. 2 f) was observed in two patients (4.3%) at final follow-up (Fig. 2 e).

## Complications

Complications included axial symptoms in three cases (6.5%). The patients with axial symptoms needed to take nonsteroidal antiinflammatory drugs orally and all relieved after 3 months. There was no C5 root palsy, adjacent segment disease and instrument failure occurred during the follow-up.

## Discussion

The optimal surgical treatment option for multilevel cervical OPLL remains controversial [1]. Surgical options include anterior corpectomy and fusion, laminoplasty, and laminectomy and fusion [5].

Anterior corpectomy and fusion allows for direct removal of the OPLL mass and is more effective at restoring cervical lordosis than posterior surgery [1]. However, disadvantages of anterior approach are technical difficulty and high complication rates which include pseudarthrosis, dysphagia, and dural tears [5]. So the posterior approaches are preferable with more than three levels involved [9].

The common posterior approaches, laminoplasty and laminectomy and fusion, use an indirect decompression with less technically demanding and lower rate of complications [10].

Laminoplasty accomplishes decompression by hinging open the laminae and results in a 30% to 40% increase in the size of canal volume [11]. Compared with laminectomy and fusion, Laminoplasty is advocated because of its preservation of neck range of motion (ROM) [5].

However, neck ROM may incite further progression of OPLL. Progression of the ossification was found in 66% of the patients underwent laminoplasty [12]. According to Yoshida, limiting cervical ROM may prevent late deterioration due to progression of OPLL [13]. Morio reported that restriction of segmental motion was associated with clinical improvement in myelopathy [14].

In addition, complications associated with laminoplasty include closure of the opened lamina, hinge fracture, development of postoperative malalignment [10]. Significant kyphosis and instability are contraindications for laminoplasty [15].

So laminectomy and fusion may be preferred for the treatment of multilevel cervical OPLL. Laminectomy and fusion removes the laminae followed by instrumented fusion and results in a 70% to 80% increase in spinal canal [11]. According to Houten, laminectomy and posterior lateral mass fusion can lead to high rates of fusion, preserved lordosis, and clinical results comparable or superior to those seen with anterior surgery [16].

A significant portion of laminectomy requires posterior fusions caudally to C6 or C7. However, cervicothoracic junction is a transition point between the lordosis of the cervical spine and the kyphosis of the thoracic spine. Furthermore, the subaxial cervical spine provides up to 20° of combined flexion/extension, 10° of lateral bending, and 5° to 7° of rotation per level. This mobility is in stark contrast to the structurally rigid thoracic spine, which permits < 5° of flexion/extension and lateral bending per level. The substantial difference between mobility in cervical and thoracic spine may amplify rates of adjacent segment disease at the cervicothoracic junction when multilevel cervical fusions are terminated in the lower cervical spine [7, 17].

So, routine extension of posterior cervical fusions into the thoracic spine has been suggested. The benefits of extension into the thoracic spine include greater surface area for the fusion mass and the larger screws typically employed in the thoracic spine which may offer greater construct rigidity and a more stable mechanical environment [18].

According to Osterhoff et al., secondary interventions due to adjacent segmental pathology or implant failure were necessary in 18/58 (31.8%) of the C7-cases and in 1/16 (6.3%) of the T1/2-cases (*p* = 0.038). So, they suggested that patients with multi-segmental posterior cervical fusions ending at C7 showed a higher rate of clinically symptomatic pathologies at the adjacent level below the instrumentation. One may consider to bridge the cervico-thoracic junction and to end the instrumentation at T1 or T2 in those cases [19].

As Schroeder et al. presented, a significant difference in the revision rates was identified between fusions terminating at C7, T1, and T2-T4 (35.3%, 18.3%, and 40.0%, *P* = 0.008). Patients whose construct terminated at C7 were 2.29 (1.16–4.61) times more likely to require a revision than patients whose construct terminated at T1 (*P* = 0.02), but no difference between stopping at T1 and T2-T4 was identified. So, they recommended that multilevel posterior cervical fusions should be extended to T1, as stopping a long construct at C7 increases the rate of revision [17].

In our study, grafted bone was completely fused in all patients. There was no adjacent segment disease and instrument failure occurred during the follow-up.

Progression of OPLL could be observed both during the natural course and after surgery. The incidence of postoperative progression reported in the literature varied from 3.3% to 74.5% [7]. As Sakai K et al. presented, postoperative progression of OPLL was observed in 5% of the anterior decompression and fusion with floating method group and 50% of the laminoplasty group [20]. Lee et al. reported that the incidence progression of OPLL was 45.5%, 62.5%, and 30% for laminoplasty, laminectomy, and laminectomy with fusion, respectively [21]. Lee et al. performed a meta-analysis of 11 studies and reported a 62.5% incidence of OPLL progression after laminoplasty and 7.6% after anterior or posterior fusion surgery [22]. So, posterior decompression with instrumented fusion surgery could suppress the progression of OPLL [7]. In our study, progression of OPLL was observed in two patients(4.3%) at final follow-up.

C5 palsy is a serious complication after cervical decompression surgery in which the patient shows deterioration in power of the deltoid or biceps brachii. According to Pan FM et al., the average incidence rate of C5 palsy after posterior cervical spine surgery was 7.8% (range, 1.4–23.0%). Risk factors for C5 palsy included age, male gender, OPLL, and stenosis of the C4–C5 intervertebral foramen [23]. Foraminotomy and intraoperative neuromonitoring were the two main methods used to prevent C5 palsy. In our study, both foraminotomy and intraoperative neuromonitoring was used to prevent C5 palsy.

Axial symptoms are defined as pain from the nuchal to the periscapular or shoulder region. According to Wang M et al., The pooled axial symptoms prevalence was 28% (95% CI 24–32). The prevalence of axial symptoms was higher after expansive open-door laminoplasty (39%) than after modified open-door laminoplasty (23%) and laminectomy instrumented fusion (29%). They suggested that postoperative axial symptoms may be reduced through preserving posterior muscles and structures, stabilizing cervical vertebrae, and reducing external cervical immobilization time [24]. The semispinalis cervicis, most of which inserts on C2, acts as a dynamic stabilizer and extensor of the cervical spine [25]. For complete preservation of the semispinalis cervicis inserted in C2, Takeuchi et al. changed the laminoplastic procedure from C3–C7 laminoplasty to C4–C7 laminoplasty with C3 laminectomy. Their report demonstrated that modified laminoplasty with preservation of the semispinalis cervicis significantly reduced postoperative axial symptoms [26]. In our study, muscle insertion of C2 spinous process was retained and cervical collar was worn for one month, which were performed to prevent axial symptoms.

C2-C7 lordosis angle and C2-C7 SVA were used to measure the translation of cervical spine in the sagittal plane [27]. Lee et al. reported expansive laminoplasty vs laminectomy alone vs laminectomy and fusion for cervical OPLL. Cervical lordosis, C2-C7 Cobb angle and CCI, decreased gradually in all patients. SVA was maintained in laminectomy and fusion group only and increased in the others [21]. According to Liu X et al., the SVA significantly increased in expansive open-door laminoplasty and was maintained in laminectomy and instrumented fusion for cases with cervical OPLL. C2-C7 Cobb angle increased in laminectomy and instrumented fusion and decreased in expansive open-door laminoplasty [28]. Our results showed that C2-C7 Cobb angle increased and C2-C7 SVA decreased, which was similar with the result of Liu X et al.

JOA was used to evaluate neurological recovery. Excellent neurological recovery is associated with the extent of decompression. Extensive laminectomy, as an alternative surgical option, allowed adequate decompression of the spinal cord and nerve roots by removing spinous processes, lamina, ligamentum flavum, and especially the inner less than 1/4 of facet joints on each side [29, 30]. According to Du W et al., enlarged laminectomy with fixation for the management of multilevel cervical degenerative myelopathy was demonstrated to be an effective strategy for improving neurological function. We performed enlarged laminectomy in patients with multilevel cervical OPLL and excellent neurological recovery was obtained.

There are several limitations in this study. Firstly, it is a retrospective, single-institution study. Then, there is no control group and sample size is small. Finally, follow-up time is insufficient. So, randomized controlled studies with long-term follow-up are needed to confirm these results. 

## Conclusion

Cervical laminectomy and crossing the cervicothoracic junction fusion for treatment of multilevel cervical OPLL is demonstrated to be an effective strategy for improving neurological function, decreasing the incidence of adjacent segment disease and instrument failure, restoring cervical lordosis, preventing progression of OPLL. Randomized controlled studies compared constructs ending at cervical vertebrae or thoracic vertebrae are needed to confirm these results.

## Data Availability

The datasets used and/or analysed during the current study are available from the corresponding author on reasonable request.

## Abbreviations

OPLL: Ossification of the posterior longitudinal ligament
SVA: Sagittal vertical axis
JOA: Japanese Orthopedic Association
SD: Standard deviation
OR: Occupying ratio
ASD: Adjacent segment disease
